# Extracorporeal membrane oxygenation: a literature review

**DOI:** 10.5935/0103-507X.20190063

**Published:** 2019

**Authors:** Renato Carneiro de Freitas Chaves, Roberto Rabello Filho, Karina Tavares Timenetsky, Fabio Tanzillo Moreira, Luiz Carlos da Silva Vilanova, Bruno de Arruda Bravim, Ary Serpa Neto, Thiago Domingos Corrêa

**Affiliations:** 1 Departamento de Medicina Intensiva, Hospital Israelita Albert Einstein - São Paulo (SP), Brasil.; 2 Departamento de Anestesiologia, Irmandade da Santa Casa de Misericórdia de Santos - Santos (SP), Brasil.; 3 Departamento de Medicina Intensiva, Hospital Municipal Dr. Moysés Deutsch - São Paulo (SP), Brasil.

**Keywords:** Extracorporeal membrane oxygenation, Respiratory insufficiency, Heart failure, Respiration, artificial, Critical care

## Abstract

Extracorporeal membrane oxygenation is a modality of extracorporeal life support that allows for temporary support in pulmonary and/or cardiac failure refractory to conventional therapy. Since the first descriptions of extracorporeal membrane oxygenation, significant improvements have occurred in the device and the management of patients and, consequently, in the outcomes of critically ill patients during extracorporeal membrane oxygenation. Many important studies about the use of extracorporeal membrane oxygenation in patients with acute respiratory distress syndrome refractory to conventional clinical support, under in-hospital cardiac arrest and with cardiogenic refractory shock have been published in recent years. The objective of this literature review is to present the theoretical and practical aspects of extracorporeal membrane oxygenation support for respiratory and/or cardiac functions in critically ill patients.

## INTRODUCTION

Extracorporeal life support is a therapeutic modality that allows for temporary support in pulmonary^(1-4)^ and/or cardiac failure^(5-9)^ refractory to conventional clinical treatment.^(1-9)^ Extracorporeal membrane oxygenation (ECMO) is one of the main extracorporeal life support devices used today.^(10)^ The venovenous ECMO (VV-ECMO) configuration is the modality of choice in cases of respiratory failure with preserved cardiac function.^(1-4)^ In turn, the venoarterial ECMO (VA-ECMO) configuration is the modality indicated to provide cardiac support in cases with preserved lung function or not.^(5-9)^

The first record of the successful use of an extracorporeal circulation device was during cardiac surgery in 1954,^(11)^ whereas the first report of the use of ECMO in the context of respiratory failure was in 1972.^(12)^ The first multicenter and randomized study that evaluated the use of ECMO in the context of respiratory failure was published in 1979.^(4)^ Since the first descriptions of ECMO, significant improvements have been made in the device, in patient management and, consequently, in the outcomes of ECMO patients.^(13)^

Several important studies of the use of ECMO in patients with acute respiratory distress syndrome (ARDS) refractory to conventional clinical support,^(1,2,14)^ patients in in-hospital cardiac arrest,^(7)^ and patients with refractory cardiogenic shock have been published in recent years.^(8)^ Thus, the objective of this literature review is to briefly present the main evidence for ECMO support in critically ill patients, as well as some practical aspects of its use.

## METHODS

This study is a nonsystematic review (narrative review) of the literature addressing theoretical and practical concepts of the use of ECMO in situations of pulmonary and/or cardiac failure refractory to conventional clinical treatment. The present review included articles published in the MEDLINE(r)/PubMed database until December 2018. The search strategy included the following terms: (("*Extracorporeal Membrane Oxygenation*" *OR* "*ECMO*") *AND* ("*systematic*" *OR* "*clinical trial*" *OR* "*random allocation*" *OR* "*therapeutic use*")). The present study identified 1,356 potentially relevant articles. After we read the titles and abstracts, 76 relevant articles were selected for a complete analysis. We also searched the reference list of the selected articles to identify other relevant studies. No language restrictions were adopted.

### Technical aspects

#### The extracorporeal membrane oxygenation circuit

**Figure 1:**
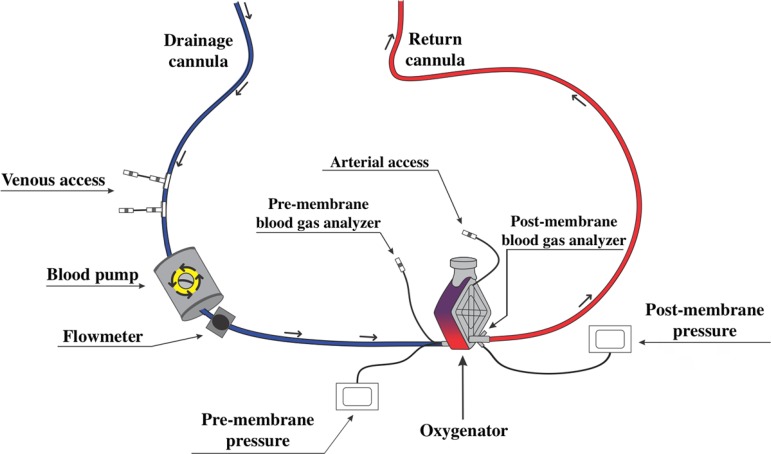
Diagram of the standard extracorporeal membrane oxygenation circuit. The venous blood is removed from the patient through a drainage cannula and is pumped (blood pump) to the oxygenator. After passing through the oxygenator, where the oxygenation membrane is, the blood is returned to the patient through an artery (venoarterial extracorporeal membrane oxygenation) or a vein (venovenous extracorporeal membrane oxygenation). There are access routes located along the extracorporeal membrane oxygenation circuit (venous and arterial access points) for infusion of medications and fluids and collection of laboratory tests, in addition to pressure sensors (pre-membrane and post-membrane) and flow sensors.

The standard ECMO circuit consists of a blood pump, oxygenator, drainage and return cannulae, flow and pressure sensors, heat exchanger for cooling or heating the blood, and arterial and venous access points for the collection of blood in the circuit (Figure 1).^(15)^

#### Blood pump

The function of the blood pump is to propel the blood of the patient to the oxygenator membrane, generating flow to the system.^(10)^ The pump is usually positioned in the line of the drainage cannula between the patient and the membrane oxygenator (Figure 1).^(15)^ Two types of pumps can be used: roller or centrifugal.^(10)^ The roller pump generates blood flow through progressive compressions of segments of the tubing of the drainage cannula, generating unidirectional and continuous blood flow.^(15)^ The centrifugal pump generates blood flow through a magnetic field generated from the rotation of an axis coupled to a disc, generating unidirectional and continuous blood flow.^(15)^ In both types of pumps, it is necessary to use safety devices that allow the system to operate in cases of power failures, such as a back-up battery and hand crank.^(10,15,16)^ The battery is activated in situations of power failure or during transport of the patient on ECMO.^(16)^ The hand crank enables the generation of blood flow if the operation of the system is not properly restored in situations of power failure.^(10,16)^

#### Oxygenator

**Figure 2:**
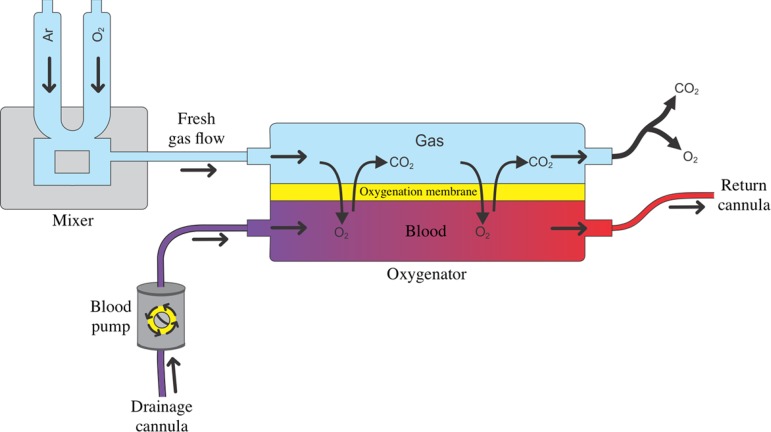
Oxygenator and oxygenation membrane. Once the cannulation of the patient is completed and the extracorporeal membrane oxygenation circuit is established, the patient's blood is pumped to the oxygenator. The oxygenator consists of a container with two chambers separated by a semipermeable membrane - the oxygenation membrane. While the patient's blood flows through one chamber, a gas mixture, called fresh gas flow, flows through the other. It is through the oxygenation membrane that gas diffusion occurs between the patient's blood and the fresh gas flow, allowing for the oxygenation of venous blood and the removal of carbon dioxide. The composition of the gas mixture in the fresh gas flow is determined by adjusting the inspired fraction of oxygen in the gas mixer. O_2_ - oxygen; CO_2_ - carbon dioxide.

The oxygenator consists of a container with two chambers separated by a semipermeable membrane, which is the oxygenation membrane, where the patient's blood flows through a chamber, while a gas mixture called the fresh gas flow flows through the other (Figure 2).^(17)^ It is through the oxygenation membrane, or oxygenator membrane, that gas diffusion occurs between the patient's blood and the fresh gas flow, allowing for oxygenation of venous blood and removal of carbon dioxide. The composition of the gas mixture in the fresh gas flow is determined by adjusting the inspired fraction of oxygen (FiO_2_) in the gas mixer (Figure 2).^(17)^ The oxygenator should preferably be made of polymethylpentene fibers because they are more efficient and long lasting than oxygenators made of polypropylene or silicone.

The partial pressure of oxygen in the blood after passing through the oxygenation membrane, or postmembrane blood, is directly proportional to the oxygen concentration in the fresh gas flow and to the blood flow that passes through the membrane. Thus, the increase in the FiO_2_ of fresh gas flow and/or the increased blood flow through the oxygenation membrane results in an increase in the oxygen concentration in the postmembrane blood.^(17)^ The concentration of carbon dioxide is mainly determined by the fresh gas flow rate, so by increasing the fresh gas flow rate, there is an increase in the removal of carbon dioxide from the blood during passage through the oxygenator membrane.^(17)^

### Modalities of extracorporeal membrane oxygenation and vascular access

**Figure 3:**
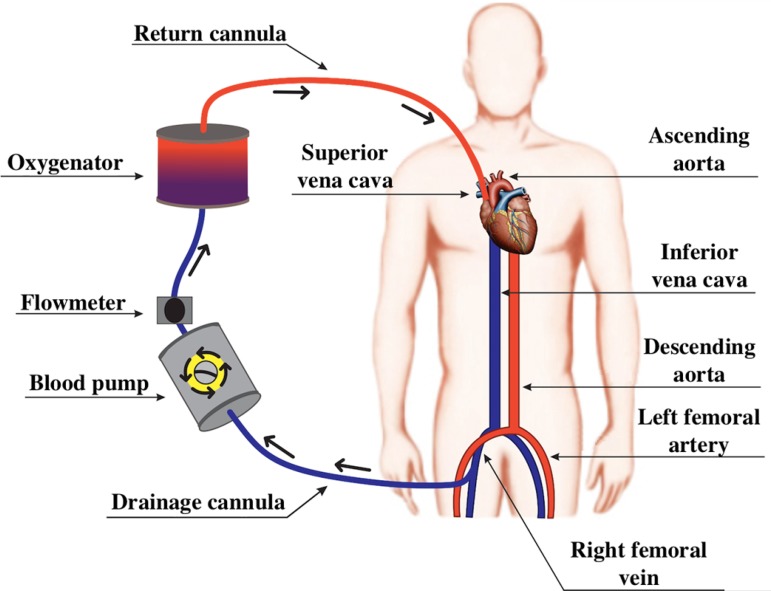
Diagram of a venovenous extracorporeal membrane oxygenation circuit. Blood from the inferior vena cava is drained through a cannula in the right femoral vein. Then, the blood passes through the propulsion pump and the oxygenation membrane, returning to the venous system of the patient through the right internal jugular vein.

**Figure 4:**
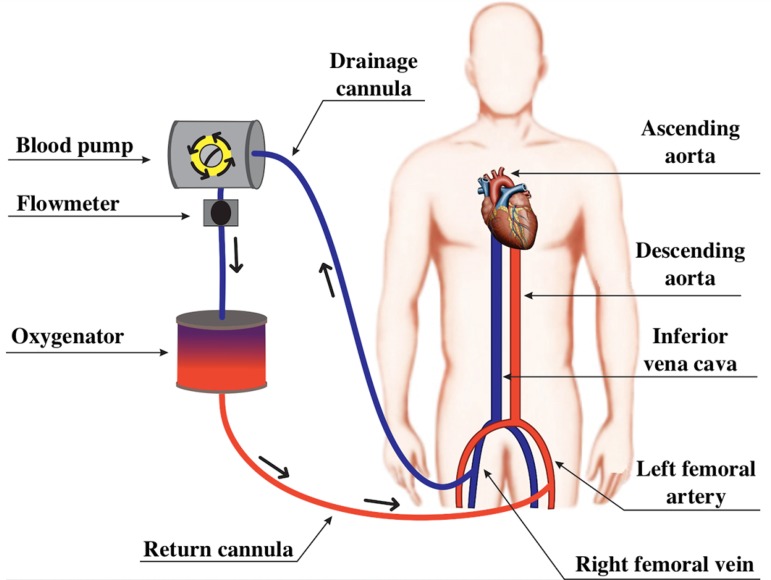
Diagram of a peripheral venoarterial extracorporeal membrane oxygenation circuit. The blood from the inferior vena cava is drained through a cannula in the right femoral vein. Then, the blood passes through the blood pump and the oxygenation membrane, returning to the arterial system of the patient through the left femoral artery.

The ECMO circuit can be configured as VV-ECMO (Figure 3) or as VA-ECMO (Figure 4).^(10,18)^ In both ECMO modalities, an access route is required for drainage, as well as an access route for return of the blood to the patient (Figures 3 and 4).^(15)^

Usually, venous accesses are performed percutaneously (Seldinger technique) and are ultrasound guided.^(16)^ The arterial accesses can be performed percutaneously or surgically.^(16)^ If using vascular access to the central vessels, right atrium or aortic artery, the surgical access (thoracotomy or median sternotomy) is the access route chosen.^(16)^ The correct placement of the cannulae, which can be confirmed by chest X-ray, ultrasound or radioscopy, is essential because it prevents complications such as inadequate blood flow, thrombus formation, structural damage to the myocardium, cardiac tamponade and recirculation phenomena, observed in VV-ECMO.^(18)^ The recirculation phenomenon consists of draining oxygenated blood through the return cannula without oxygenated blood passing through the systemic circulation. To minimize the occurrence of the recirculation phenomenon, the femoral venous drainage cannula should ideally be placed at the level of the suprahepatic inferior vena cava, maintaining a minimum distance between the distal extremities of the drainage and return cannulae greater than 10cm.^(16)^

The drainage and return cannulae can be made of wire or plastic. Wire cannulae are preferably used because they are less likely to bend, especially during the movement of patients.^(10,15)^ In adult patients, the venous cannulae used usually have a length of 50 to 70cm and a diameter of 19 to 25Fr, and they are multiperforated at the distal end. The arterial cannulae are usually smaller than the venous cannulae, with lengths between 20 and 40cm, diameters of 17 to 22Fr and distal orifices, in combination or not with lateral orifices.^(10,15)^ The diameter of the drainage cannula is especially important because the resistance to blood flow is inversely proportional to the diameter of the return cannula.^(10,15)^ Therefore, cannulae with the largest possible diameter should be used to optimize blood flow.^(10,15)^

In VV-ECMO, the drainage cannula is usually inserted into the right femoral vein and the return cannula into the right internal jugular vein (Figure 3).^(19)^ Alternatively, the drainage cannula can be inserted into the jugular vein and the return cannula into the femoral vein.^(19)^ The use of a double lumen cannula, which is not yet available in Brazil, allows for blood drainage and return functions to occur through the same venous access, affording greater patient mobility.

In VA-ECMO, the drainage cannula is inserted into a venous access and the return cannula into an arterial access, and VA-ECMO can be classified as central or peripheral, according to the cannulated vessels (Figure 4).^(15)^ In the central configuration, the drainage cannula can be inserted directly into the right atrium and the return cannula into the ascending segment of the aorta.^(15)^ In the peripheral configuration, blood can be drained through the femoral or jugular veins, and it returns to the patient through the carotid, axillary or femoral arteries (Figure 4).^(15)^ Thus, a characteristic of VA-ECMO is the exclusion of pulmonary circulation.^(15)^

It is recommended that, immediately before peripheral or central canalization, an heparin bolus of 50 to 100 U/kg be administered since the blood flow can be reduced or absent during cannulation, favoring the formation of clots.^(16)^

### Indications for extracorporeal membrane oxygenation

Indications for ECMO can be divided into four categories: hypoxemic respiratory failure, hypercapnic respiratory failure, cardiogenic shock and cardiac arrest.^(2,20,21)^ The main indications for ECMO are presented in table 1.

**Table 1:** Indications for extracorporeal membrane oxygenation

Hypoxemic respiratory failure (primary or secondary cause)
PaO_2_/FiO_2_ < 100, with FiO_2_ > 90% and/or Murray score 3 - 4 for more than 6 hours
PaO_2_/FiO_2_ < 80, with FiO_2_ > 80% for more than 3 hours
Bridge to lung transplantation
Hypercapnic respiratory failure
pH ≤ 7.20 with RR of 35rpm, tidal volume of 4 - 6mL/kg of predicted weight and DP ≤ 15cmH_2_O
Bridge to lung transplantation
Heart failure
Cardiogenic shock associated with acute myocardial infarction
Fulminant myocarditis
Septic shock-associated myocardial depression
Extracorporeal cardiopulmonary resuscitation
Cardiogenic shock post-cardiotomy or post-heart transplant
Post-heart transplant graft failure
Bridge to implantation of a ventricular assist device
Bridge to heart transplantation

PaO_2_ - partial pressure of oxygen in arterial blood; FiO_2_ - fraction of inspired oxygen; RR - respiratory rate; DP - distending pressure.

VV-ECMO is preferentially used in patients with preserved or moderately reduced cardiac function, being the modality of choice in patients with hypoxemic respiratory failure and hypercapnic respiratory failure (Figure 3).^(21,22)^ The main clinical trials that evaluated the use of VV-ECMO in patients with ARDS are summarized in table 2.^(1-4)^ The VA-ECMO configuration is indicated for patients with heart failure for whom pulmonary support might or might not be necessary (Figure 4).^(22)^ The main clinical trials that evaluated the use of VA-ECMO are summarized in table 3.^(5,7-9)^

**Table 2:** Characteristics of the main studies that evaluated the use of venous extracorporeal membrane oxygenation in patients with acute respiratory distress syndrome

Author	N	Design	Inclusion criteria	Exclusion criteria	Primary outcome	Main findings	Considerations
Combes et al.^(1)^	249	International multicenter, randomized, controlled clinical trial	1. Patient intubated on MV < 7 days 2. PaO_2_/FiO_2_ < 50mmHg for > 3 hours OR PaO_2_/FiO_2_ < 80mmHg for > 6 hours OR Arterial pH < 7.25 with PaCO_2_ ≥ 60mmHg > 6 hours 3. Optimized MV* 4. Age >18 years	1. Pregnant women 2. BMI > 45 3. Chronic respiratory failure 4. Indication for VA-ECMO 5. History of HIT 6. Advanced cancer 7. Dying patients 8. Coma after cardiac arrest 9. Nonreversible neurologic injury 10. Palliative patients	60-day mortality of 35% (44/124 patients) in the ECMO group and 46% (57/125 patients) in the control group (RR: 0.76; 95%CI 0.55 -1.04; p = 0.09)	The ECMO group had a higher incidence of severe thrombocytopenia and bleeding requiring transfusion. The ECMO group had a lower incidence of IS, lower need for renal replacement therapy, and reduction of tidal volume, plateau pressure and drive pressure	Early interruption of the study due to futility Slow recruitment rate High *crossover* rate (28%) from the control group to the ECMO group due to refractory hypoxemia Greater failure of treatment at 60 days in the control group
Peek et al.^(2)^	180	Multicenter, randomized, controlled clinical trial	1. Age from 18 - 65 years 2. Severe but potentially reversible respiratory failure 3. Murray score ≥ 3.0 4. Uncompensated hypercapnia 5. Optimized MV	1. PIP > 30cmH_2_O 2. FiO_2_ > 80% 3. Time of MV ≥ 7 days 4. Intracranial bleeding 5. Contraindication to heparinization 6. Limitation of support	6-month mortality after randomization or before hospital discharge of 37% (33/90) in the ECMO group and 53% (46/87) in the control group (RR: 0.69; 95%CI 0.05-0.97, p = 0.03)	The transfer of patients with severe but potentially reversible respiratory failure to a reference center in ECMO proved to be cost-effective and reduce mortality	Control group does not have standardization of MV parameters Of the 90 patients randomized to receive ECMO, 22 patients did not use the device
Morris et al.^(3)^	40	Dual-center, randomized, controlled clinical trial	1. PaO_2_ < 50mmHg for 2 hours with FiO_2_ = 100%, PEEP > 5 and PaCO_2_ of 30 - 45 or PaO_2_ < 50mmHg for 12 hours with FiO_2_ = 60%, PEEP ≥ 5cmH_2_O and PaCO_2_ of 30 - 45 2. Optimized MV	1. Contraindication to anticoagulants 2. POAP > 25mmHg 3. Time of MV> 21 days 4. Severe, irreversible and without treatment prospective systemic disease.	30-day survival of 33% (7/21) in the ECMO group and 42% (8/19) in the control group (p = 0.8)	Does not recommend the use of ECMO in patients with ARDS	Small sample size High mortality rate (62% of patients died) Technical limitations inherent to the clinical trial period Nonprotective MV in both groups
Zapol et al.^(4)^	90	Multicenter, randomized, controlled clinical trial	1. PaO_2_ < 50 mm Hg, for more than 2 hours with FiO_2_ 100% and PEEP ≥ 5cmH_2_O OR PaO_2_ < 50 mm Hg, for more than 12 hours with FiO_2_ = 60% and PEEP ≥ 5cmH_2_O	1. Age from 12 to 65 years old 2. Pulmonary lesion time > 21 days 3. PWP > 25mmHg 4. Severe, irreversible and incurable systemic disease.	30-day survival of 9.5% (4/42) in the ECMO group and 8.3% (4/48) in the control group (no significant difference)	ECMO was able to provide respiratory support, but did not increase the survival in patients with severe ARDS	Mortality in both groups greater than 90% Technical limitations inherent to the clinical trial period Nonprotective MV in both groups

MV - mechanical ventilation; PaO_2_ - partial pressure of oxygen; FiO_2_ - fraction of inspired oxygen; PaCO_2_ - partial pressure of carbon dioxide; BMI - body mass index; VA-ECMO - venoarterial extracorporeal membrane oxygenation; HIT - heparin-induced thrombocytopenia; RR - relative risk; 95%CI - 95% confidence interval; IS - ischemic stroke; PIP - peak inspiratory pressure; PEEP - positive end-expiratory pressure; PWP - pulmonary wedge pressure; ARDS - acute respiratory distress an inspired fraction of oxygen ≥ 0.80 syndrome.

* Defined by the presence of, positive end-expiratory pressure ≥ 10cmH_2_O and tidal volume of 6mL/kg of predicted weight.

**Table 3:** Characteristics of the main studies that evaluated venoarterial extracorporeal membrane oxygenation in patients with refractory cardiogenic shock and/or in-hospital cardiac arrest.

Author	N	Design	Inclusion criteria	Exclusion criteria	Primary outcome	Main findings	Conclusion
Dangers et al.^(5)^	105	Retrospective analysis, single center	Patients who used VA-ECMO due to cardiogenic shock with dilated cardiomyopathy	Refractory cardiogenic shock due to complications of acute heart disease (myocardial infarction and myocarditis) Patients who used VV-ECMO	Description of characteristics, outcomes and risk factors associated with worse outcomes in patients on VA-ECMO due to cardiogenic shock	One-year survival of 42% One-year survival of patients with pre-VA-ECMO SOFA < 7 was 52%. One-year survival of patients with pre-VA-ECMO SOFA > 13 of 17% 67% of patients used IABP in combination with VA-ECMO.	VA-ECMO as a bridge to left ventricular assist device or heart transplantation should be considered in patients with cardiogenic shock VA-ECMO is best indicated in patients with SOFA < 11
Rastan et al.^(6)^	517	Observational prospective study	Patients who used VA-ECMO for refractory cardiogenic shock after cardiotomy	Not specified	Identification of risk factors associated with hospital outcomes and long-term outcomes	Six-month survival of 17.6% One-year survival of 16.5% Five-year survival of 13.7% Age > 70 years, diabetes, renal failure prior to surgery, obesity, lactate > 4 mmol/L are risk factors for in-hospital mortality	VA-ECMO is an acceptable option for patients with refractory cardiogenic shock after cardiotomy
Chen et al.^(7)^	172	Observational prospective study, single center. Matching performed with propensity score	Intrahospital cardiac arrest Age between 18 and 75 years Cardiac arrest lasting > 10 minutes	Previous irreversible neurological disease Terminal stage cancer Uncontrolled bleeding of traumatic origin	Survival to hospital discharge in the ECMO group of 28.8% (17/59) and 12.3% (14/113) in the control group (*log-rank* p < 0.0001)	Return to spontaneous circulation was higher in the ECMO group. One-year survival in the ECMO group was 18.6% (11/59) One-year survival in the control group was 9.7% (11/113).	VA-ECMO in in-hospital cardiac arrest increased survival and improved neurological outcomes compared to conventional CPR
Combes et al.^(8)^	81	Retrospective study	Patients who used VA-ECMO for refractory cardiogenic shock	Patient using VV-ECMO	Identification of early and independent predictors of ECMO failure and description of the outcome of patients on ECMO support during ICU stay	Variables associated with increased mortality: onset of ECMO during cardiac arrest, severe hepatic or renal dysfunction and female sex ECMO due to fulminant myocarditis was associated with better outcomes	VA-ECMO in patients with refractory cardiogenic shock is effective in rescue in 40% patients Survival in the ICU in the ECMO group was 42% (34/81).
Pagani et al.^(9)^	33	Not specified	Absence of contraindication to heart transplantation Age < 66 years Refractory cardiogenic shock Severe hemodynamic instability	Need for VA-ECMO after transplant failure Elective and planned use of VA-ECMO for coronary angioplasty	Evaluation of the use of ECMO as a bridge to LVAD and subsequent transplantation in selected high-risk patients	Small sample size VA-ECMO is effective in the initial stabilization of patients with refractory cardiogenic shock, but maintenance of VA-ECMO is associated with a high rate of complications The cost of VA-ECMO compared to the LVAD was lower but had a higher incidence of complications	The initial stabilization of patients with refractory cardiogenic shock with VA-ECMO as a bridge to LVAD or heart transplantation is associated with better outcomes at 1 year.

VA-ECMO - venoarterial extracorporeal membrane oxygenation; VV-ECMO - venovenous extracorporeal membrane oxygenation; SOFA - Sequential Organ Failure Assessment Score; IABP - intra-aortic balloon pump; LVAD - left ventricular assist device; CPR - cardiopulmonary resuscitation; ICU - intensive care unit.

#### Indications for venovenous extracorporeal membrane oxygenation

The indications for VV-ECMO are classically divided into hypoxemic respiratory failure and hypercapnic respiratory failure. The Extracorporeal Life Support Organization (ELSO) report showed that the three main indications for VV-ECMO [mean ECMO duration (hour)]; survival (%)] are: bacterial pneumonia (261 hours; 61%), viral pneumonia (325 hours; 65%) and postoperative or trauma-related to ARDS (256 hours; 57%).^(23)^

Brazilian mechanical ventilation guidelines indicate the use of VV-ECMO in cases of refractory hypoxemia, defined as the ratio between the partial pressure of oxygen and FiO_2_ (PaO_2_/FiO_2)_ < 80, with FiO_2_ > 80% after performing adjuvant and rescue maneuvers for severe ARDS for at least 3 hours. In cases of hypercapnic respiratory failure, the Brazilian guidelines establish as criteria for the use of ECMO the presence of hypercapnia with pH ≤ 7.20, a respiratory rate (RR) of 35rpm and tidal volume between 4 and 6mL/kg of predicted weight, and a mandatory distending pressure ≤ 15cm H_2_O.^(24)^ Because this guideline is a national standard of conduct, its adoption is suggested for the indication of VV-ECMO in cases of hypoxemic or hypercapnic respiratory failure.^(24)^ The characteristics and outcomes of the main studies that evaluated the use of VV-ECMO in patients with ARDS are shown in table 2.

### Indications for venoarterial extracorporeal membrane oxygenation

Venoarterial ECMO is indicated in cases of cardiogenic shock, in which the patient has low cardiac output and tissue hypoperfusion, despite hemodynamic optimization with volume replacement, use of inotropes, vasopressors or vasodilators and/or intra-aortic balloon pump counterpulsation.^(25-27)^ The ELSO report showed that the four main indications for VA-ECMO [mean ECMO duration (hour)]; survival (%)] are: cardiogenic shock (144 hours; 42%), cardiomyopathy (162 hours; 51%), congenital heart disease (129 hours; 37%) and myocarditis (188 hours; 65%).^(23)^ Characteristics and outcomes of the main studies evaluating VA-ECMO in patients with refractory cardiogenic shock and/or in-hospital cardiac arrest are shown in table 3.

### Initial adjustments of extracorporeal membrane oxygenation

In VV-ECMO, an initial blood flow by the system of 50mL/kg/minute of ideal body weight is suggested, which is then adjusted to maintain the peripheral saturation of hemoglobin measured by pulse oximetry (SpO_2_) > 80%.^(22)^ In addition to this initial value, a determinant factor for hypoxemia correction is the ratio between the system flow and native cardiac output, and system flow values of approximately 60% of cardiac output are required to ensure the desired systemic oxygenation, i.e., SpO_2_ > 80%.

In VA-ECMO, an initial blood flow by the system of 30 mL/kg/minute of ideal body weight is suggested and then is adjusted so that the central venous oxygen saturation is > 70%.^(16)^

The fresh gas flow should be adjusted to maintain the pH at close to 7.40 and the partial pressure of carbon dioxide (PaCO_2_) at 40mmHg, and in a patient with PaCO_2_ > 50mmHg, the reduction must be slow and gradual, not exceeding reduction values greater than 10mmHg per hour.^(22)^ In a patient with an indication for ECMO due to hypercapnia, it is suggested that initially the blood flow be low (1L/minute) and the fresh gas flow high (15L/minute), with subsequent adjustment with the goal of maintaining the pH at values at close to 7.40 and PaCO_2_ at values close to 40mmHg.^(22)^

### Mechanical ventilation in extracorporeal membrane oxygenation

Patients with ARDS on ECMO should be ventilated in a protective manner, avoiding lung injury induced by the mechanical ventilator. Patients should be ventilated with a low tidal volume, low FiO_2_, and low plateau pressure and peak pressure.^(28,29)^

The ELSO consensus recommends that patients be sedated to a moderate or deep level and ventilated in the first 24 hours of support with ECMO with low RR (5rpm), with inspiratory-to-expiratory time 2:1, plateau pressure < 25cmH_2_O, FiO_2_ of 50%, and positive end-expiratory pressure (PEEP) of 15cmH_2_O in a pressure-controlled ventilation mode.^(22)^ Between 24 and 48 hours after the start of ECMO support, if the patient is hemodynamically stable, it is recommended that the plateau pressure be reduced to 20cmH_2_O, FiO_2_ to 21 to 40%, and PEEP to 10cmH_2_O.^(22)^ After 48 hours of ECMO, if the patient remains stable and with hemodynamic improvement, it is recommended that sedation be minimal.^(22)^

Another strategy for initial PEEP adjustment can be performed according to the CESAR study.^(2)^ PEEP is initially adjusted to 10 cm H_2_O and then adjusted to the best SpO_2_ value.^(2,30)^ Electrical impedance tomography can be used at the bedside as a tool for titrating PEEP.^(31)^ The titration of PEEP aims to improve pulmonary compliance and oxygenation and to reduce the difference between plateau pressure and PEEP, minimizing the risk of atelectotrauma and biotrauma.^(32)^

The ideal tidal volume for patients with ECMO is not consensual.^(32)^ Usually, a tidal volume of approximately 4mL/kg of predicted weight is adopted,^(28,29,32)^ with a tidal volume of less than 1.5mL/kg of predicted weight being described in a patient with VV-ECMO.^(33)^ In our institution, we usually chose to use the following mechanical ventilation configuration: tidal volume 4 - 6mL/kg of predicted weight; PEEP initially between 10 and 15cmH_2_O (then titrated at the bedside, with electrical impedance tomography); plateau pressure ≤ 25cmH_2_O; RR of 10 ventilation cycles per minute; and the lowest FiO_2_ possible to achieve the desired PaO_2_.

### Contraindications

The ELSO consensus defines that there is no absolute contraindication to the use of ECMO, but the risk is such that the benefit of ECMO support should be individualized for each patient.^(22)^ However, there are situations in which the benefit of the ECMO is questionable and is considered a contraindication to its use. The main contraindications include uncontrolled active hemorrhage, incurable cancer, solid organ transplant or immunosuppression, irreversible central nervous system dysfunction, and irreversible or terminal heart or respiratory failure in patients who are not transplant candidates.^(20,21)^

### Complications

Complications during the management of patients on ECMO are frequent.^(34)^ A retrospective analysis of 265 adult patients with ARDS showed that 31% of patients required at least one replacement of the ECMO system due to technical problems (worsening of gas exchange, coagulation disorders induced by the device and suspicion of infection in the ECMO circuit), and among the patients who needed a replacement, 45% were in urgent need of one.^(34)^ Additionally, the most common complications were progressive clot formation in the oxygenator membrane (51%), sudden clot formation in the oxygenator membrane or in the blood pump (35%) and acute mechanical failure of the ECMO system (10%).^(34)^ The main complications reported during ECMO are failure of the oxygenation membrane, rupture of the circuit, coagulation of the system, intracranial hemorrhage, acute kidney injury (AKI) and infections. The main laboratory tests used for the management of patients on ECMO are summarized in table 4.

**Table 4:** Main laboratory tests used for the management of patients on extracorporeal membrane oxygenation

Exams	When to collect	Therapeutic target	Considerations
ACT	Immediately after cannulation of ECMO	Initially, between 180 and 220 seconds. After collection of test, anticoagulation adjustment should be guided by aPTT or anti-Xa activity.	Easy to perform, can be performed at the bedside Result available quickly It allows the initial adjustment of heparin infusion
aPTT	Daily. It can be collected more than once per day, especially in cases of adjustment of heparin infusion.	Keep between 40 and 55 seconds	Adequate management of anticoagulation is essential to avoid complications such as system coagulation and intracranial hemorrhage.
Anti-Xa activity	Alternative to the aPTT. It can be collected more than once per day, especially in cases of adjustment of heparin infusion.	Keep between 0.2 and 0.3IU/mm	Adequate management of anticoagulation is essential to avoid complications such as system coagulation and intracranial hemorrhage.
Platelets	Daily. It can be collected more than once per day, especially in cases of bleeding.	Ideally kept greater than 100,000 cells per mm^3^	Platelets are an essential component of hemostasis and in the prevention of hemorrhagic complications.
Hemoglobin	Daily. It can be collected more than once per day, especially in cases of bleeding.	Ideally kept greater than 8.0 g/dL	Hemoglobin is a key component of oxygen transport.
D-Dimer	Daily. It can be collected more than once per day	Not applicable	Sudden elevation of D-dimer level is strongly indicative of clot formation, predicting failure of the ECMO system.
SvcO_2_	Daily	Ideally maintained greater than 70%, especially in VA-ECMO.	It allows for the adjustment of VA-ECMO flow.
PaCO_2_	Daily	Ideally, it was maintained close to 40mmHg, especially in VV-ECMO.	Allows for the adjustment of the fresh gas flow rate Patients with PaCO_2_ > 50mmHg should be subjected to slow and gradual reduction (not to exceed reduction values greater than 10mmHg per hour).

ACT - activated coagulation time; ECMO - extracorporeal membrane oxygenation; aPTT: activated partial thromboplastin time; VA-ECMO - venoarterial extracorporeal membrane oxygenation; VV-ECMO - venovenous extracorporeal membrane oxygenation. It is recommended that heparin infusion be initially guided by the activated coagulation time. After the collection of laboratory tests, heparin infusion should ideally be guided by the activated partial thromboplastin time or, alternatively, by anti-Xa activity.

### Failure of the oxygenation membrane

The proper functioning of the oxygenation membrane is essential for the success of ECMO.^(15)^ Regular inspection of the entire ECMO circuit and of the oxygenation membrane should be performed for early identification of factors that can compromise its operation.^(10)^ The incidence of failure of the oxygenation membrane in adult patients is 9.1% in VV-ECMO and 6.6% in VA-ECMO.^(23)^

The main reason for failure of the oxygenation membrane is the formation of clots. Detailed visual inspection of the system usually allows for the identification of forming clots.^(10,35)^ In addition to visual inspection, monitoring of the system should evaluate possible indicators of failure in the oxygenation membrane, such as a drop in the partial pressure of post-oxygenator oxygen, increasing transmembrane pressure gradients, a progressive increase in the fresh gas flow and sudden increases of D-dimer levels.^(10,36)^ The D-dimer level can be measured daily to monitor clot formation and degradation and to predict the development of oxygenation membrane failure (Table 4).^(36)^ A sudden increase in the D-dimer level is strongly indicative of clot formation and a predictor of failure of the ECMO system.^(36)^ Circuit thrombosis usually occurs in places with low flow or turbulent flow.^(34,37)^ The main factors that increase the risk of circuit thrombosis are inadequate anticoagulation, the presence of disseminated intravascular coagulation, heparin-induced thrombocytopenia and antithrombin III deficiency.^(34,37)^

### Rupture of the extracorporeal membrane oxygenation circuit

Fissures or ruptures of the ECMO circuit can occur in all components of the system.^(15,34)^ Gas embolisms and tubing ruptures or disconnections are complications that can require immediate discontinuation of ECMO.^(15)^ Ruptures in the circuit after the pump, in which the circuit is under positive pressure, can lead to rapid exsanguination of the patient.^(16,20)^ In case of cracks or ruptures of the circuit that occur after the pump, the pump should be immediately clamped, temporarily interrupting the circulatory support while the component is replaced.^(16,20)^ In cases of fractures or ruptures in the venous circuit, which is under negative pressure generated by the centrifugal pump, there is a risk of gas embolism.^(15)^ All cracks require the replacement of part of or the entire circuit, depending on the rupture site and the availability of individual system components for replacement.

### Management of anticoagulation and coagulation of the system

The main complication of VV-ECMO is the formation of clots in the system.^(38)^ One of the main challenges in the management of patients on ECMO is establishing a balance between hemostasis and thrombosis.^(34)^ Thus, clinical and laboratory monitoring of coagulation factors should be performed daily (Table 4).^(34)^

The ELSO anticoagulation guidelines recommend that, in patients who are candidates for ECMO, if there is time and availability of resources, hemostasis should be evaluated before the beginning of ECMO with the following tests: complete blood count, prothrombin time, fibrinogen, D-dimer, antithrombin, and thromboelastography or thromboelastometry.^(39)^ Thus, the identification and correction of hemostasis disorders before the beginning of ECMO can facilitate the management of anticoagulation during ECMO.^(39)^

Intravenous unfractionated heparin is the gold standard for anticoagulation therapy of patients on ECMO due to its low cost, easy titration, bedside monitoring and the possibility of reversal with protamine.^(34,40)^ It is recommended that the heparin infusion be initially guided by the activated coagulation time (ATC) and, after collection of laboratory tests, it should ideally be guided by the activated partial thromboplastin time (aPTT) or anti-Xa activity (Table 4).^(30,34)^

Intravenous unfractionated heparin infusion is usually initiated at a dose of 7.5 to 20 units/kg/hour, and the initial goal is to maintain the ATC in the therapeutic range between 180 and 220 seconds.^(30,34,39)^ Therapeutic anticoagulation is usually achieved with infusion of intravenous unfractionated heparin at a dose of 20 to 50 units/kg/hour.^(39)^ Subsequent adjustments to unfractionated heparin infusion aim to maintain aPTT at between 40 and 55 seconds and/or anti-Xa activity at between 0.2 and 0.3IU/mm of blood.^(1)^

The cutoff values for transfusion of blood products lack more robust evidence in the literature.^(39)^ In our center, we perform platelet transfusion to maintain a platelet count greater than 50,000 cells per mm^3^ in patients with active bleeding and greater than 20,000 cells per mm^3^ in patients without active bleeding. We aim to maintain a hemoglobin concentration greater than 8.0g/dL and a fibrinogen concentration greater than 100mg/dL (Table 4).

### Intracranial hemorrhage

Intracranial hemorrhage is a dreaded complication in patients on ECMO because of the complex management between intracranial bleeding control and the adjustment of anticoagulation required to maintain ECMO.^(15)^ The pathophysiology of intracranial hemorrhage, in the context of ECMO, is uncertain, and proper management of anticoagulation is essential to reduce the incidence of intracranial hemorrhage.^(40)^ According to the ELSO report, the incidence of intracranial hemorrhage and ischemic stroke in adult patients on VV-ECMO is 2.2% and 3.8%, respectively.^(23)^ However, the true incidence of intracranial hemorrhage in patients on ECMO is unknown, given the greater difficulty in diagnosing neurological events in patients on ECMO.^(15,41)^

### Acute kidney injury

The pathophysiological mechanism of AKI in patients on ECMO remains uncertain.^(42)^ It is believed that the systemic inflammatory response, intravascular volume depletion, arterial hypotension, tissue hypoperfusion and hemolysis during ECMO are involved in the pathophysiology of AKI in these patients.^(42)^ AKI developing during ECMO is associated with increased mortality and costs, with oliguria and hypervolemia being the main indications for acute hemodialysis in patients on ECMO.^(43)^

For patients refractory to clinical measures, such as diuretics and water restriction, and those requiring renal replacement therapy, the modality of choice is continuous renal replacement therapy.^(16,44)^ The optimal time to start renal replacement therapy, whether early or late, is controversial in the general population^(45)^ and in patients on ECMO.^(44)^ To date, there is no consensus regarding whether the time of onset of renal replacement therapy, early or late, reduces the risk of mortality.^(44,45)^ Early onset enables the rapid control of blood volume, acid-base balance, and water and electrolyte disorders.^(44,45)^ Late onset could allow for the recovery of renal function, avoiding complications of the vascular access (pneumothorax and catheter-related bloodstream infection) or renal replacement therapy (hypotension, hypothermia and reduction of serum levels of drugs).^(44,45)^

The ELSO consensus reports that the incidence of AKI in adult patients on VV-ECMO and VA-ECMO is 9.3% and 12.3%, respectively.^(23)^ The development of AKI requiring renal replacement therapy in patients on VA-ECMO is associated with a significant increase in mortality (odds ratio = 8.95; 95% confidence interval = 1.4 - 45.7).^(46)^ Thus, the ELSO consensus not recommend the begin of VA-ECMO in patients with kidney failure.^(25)^

### Infectious complications

Extracorporeal membrane oxygenation is an additional risk factor for the development of infection.^(10,47)^ Patients on ECMO are commonly using multiple invasive devices, such as a pulmonary artery catheter, invasive blood pressure measuring device and central venous catheter, increasing the risk of bloodstream infection, which is directly proportional to ECMO duration.^(47)^ The diagnosis of infection in a patient on ECMO can be difficult since clinical signs and classic symptoms associated with nosocomial infection, such as fever and leukocytosis, might not be present.^(10,48)^ The patient might be unable to raise the body temperature and might present with fever, mainly due to heat loss from the ECMO circuit.^(10)^ The ECMO patient often has increased leukocytes secondary to extracorporeal circulation because the blood circulates through the non-epithelialized ECMO circuit, triggering an inflammatory response.^(10)^

Due to the difficulty in establishing the diagnosis of infection, many centers use a routine surveillance culture in patients on ECMO. However, the use of prophylactic antibiotics during ECMO is not recommended.^(10)^ It is noteworthy that, with the goal of reducing thrombogenesis and increasing biocompatibility, the ECMO circuit is coated with bioactive and biopassive products. Thus, the ECMO circuit coating can allow for the adsorption of lipophilic drugs, reducing their bioavailability and rendering the biodistribution and dosage of antibiotics in patients on ECMO uncertain.^(38)^ Thus, the ELSO consensus recommends the use of appropriate antibiotics in cases of documented infection.^(16)^ The ELSO report estimates that the incidence of infection in adult patients is 17.5% in VV-ECMO and 13.0% in VA-ECMO,^(23)^ and the pathogens commonly associated with bloodstream infection include *Candida* (12.7%), *Pseudomonas aeruginosa* (10.5%) and *Staphylococcus aureus* (9.4%).^(47)^

### Weaning from extracorporeal membrane oxygenation

Removal of ECMO support is conditioned on the improvement of organ dysfunctions and resolution of the indication for support with ECMO.^(16)^ Weaning from VV-ECMO, due to acute, hypoxemic or hypercapnic respiratory failure, can be initiated when the patient is able to satisfactorily maintain gas exchange with acceptable mechanical ventilation parameters (peak pressure ≤ 30cmH_2_O, PEEP ≤ 15cmH_2_O, tidal volume ≤ 6mL/kg of predicted weight, RR ≤ 35rpm and FiO_2_ ≤ 60%), in combination with improved radiographic parameters and pulmonary compliance.^(30)^ At our institution, we perform a spontaneous breathing test for weaning from VV-ECMO, which consists of interrupting the fresh gas flow from the system. During the spontaneous breathing test, it is essential that the respiratory and hemodynamic parameters, such as SpO_2_, RR, end-tidal carbon dioxide (EtCO_2_), heart rate and mean arterial pressure, be rigorously monitored. In patients who remain stable during the autonomy test for up to 6 hours, we perform an arterial blood gas analysis. If the pH and PaO_2_ are within the target range, we consider the removal of the VV-ECMO support.

Weaning from VA-ECMO depends on the improvement of cardiac function.^(16)^ Predictors that indicate cardiac function recovery include the maintenance of continuous arterial pulse pressure for at least 24 hours, echocardiography with evidence of recovery of systolic function (ejection fraction of the left ventricle ≥ 20%) and adequate arterial oxygenation.^(8)^ The most traditional approach for VA-ECMO weaning consists of the gradual and progressive reduction of the pump flow until the contribution of the circuit to oxygenation and/or cardiac output of the patient is negligible, usually with pump flow values less than 1L/minute. Then, the arterial and venous circuits are clamped for 1 to 2 minutes.^(8)^ The hemodynamic parameters should be rigorously monitored, and the patient should remain stable during the spontaneous breathing test. It is recommended that the echocardiogram be repeated after the ECMO circuit is clamped. If the cardiac index is maintained at higher than 2.2L/min/m^2^, with ventricular ejection fraction > 35%, and the patient remains stable for at least 24 hours, VA-ECMO can be removed.^(8)^ If there is the impossibility of VA-ECMO removal, the use of a ventricular assist device, such as a bridge-to-transplantation, should be considered.^(8)^ Ideally, removal of the VA-ECMO cannula should be performed 30 to 60 minutes after discontinuation of heparin.^(16)^ The venous cannulae can be removed at the bedside, and the arterial cannulae are usually removed in the operating room.

### Role of the multidisciplinary team in extracorporeal membrane oxygenation management

Complications during the management of patients on ECMO are frequent, and a trained and engaged multidisciplinary team is crucial for the proper management of patients on ECMO, including prevention, early recognition and adequate treatment of complications when present.

The multidisciplinary team must be able to recognize the main complications, such as failure of the oxygenation membrane, rupture of the circuit, coagulation of the system, AKI and infection. Additionally, the multidisciplinary team should ideally participate in the titration of vasoactive drugs, sedation and analgesia protocols, adjustment of anticoagulation guided by specific targets, collection of laboratory tests, mobilization of the patient and prevention of pressure ulcers, in addition to providing psychosocial support to the relatives of the patient on ECMO. The periodic inspection of the circuit by the multidisciplinary team is crucial. Circuit inspection aims to monitor its integrity, evaluate the presence of clots and gas bubbles, and measure the transmembrane pressure gradient.^(10,34)^ It is part of the visual inspection, for example, to check whether there are dark or white areas in the oxygenator membrane or connections suggestive of coagulation of the system.^(10,34)^

The high complexity and numerous peculiarities of patients on ECMO are noteworthy, and the creation of continuing education programs and specific ECMO training is essential. Thus, a trained and engaged multidisciplinary team is essential for the safety of patients on ECMO because the clinical outcomes of these patients are directly related to the center's experience in the management of these patients.

### Extracorporeal membrane oxygenation in Brazil

The use of ECMO as a therapeutic modality in Brazil is a relatively recent practice. Although the first randomized study on VV-ECMO in patients with ARDS was published in 1979,^(4)^ only in 2017 did the Federal Council of Medicine (*Conselho Federal de Medicina* - CFM) stop considering ECMO to be an experimental procedure. According to opinion 42/2017 of the CFM, ECMO was recognized as a nonexperimental procedure of high risk and complexity. Currently, Brazil has 7 cities and 13 centers accredited by ELSO,^(49)^ but it does not have protocols for the transfer of candidate patients for ECMO to reference centers. The objective of transferring candidate patients for ECMO to reference centers consists of the rational use of resources allocated to health, in addition to improving the outcomes of critically ill patients in Brazil. In Brazil, the estimated current equipment cost per patient ranges from US $10,000 to US $30,000.00. Despite the high cost, it has been demonstrated in international cost-effectiveness analyses that ECMO, when properly indicated, is cost-effective, justifying the investment.^(2)^

## CONCLUSION

Extracorporeal membrane oxygenation is one of the main extracorporeal life support devices currently used in critically ill patients, allowing for temporary support in pulmonary and/or cardiac failure refractory to conventional clinical management. It is essential that physicians, nurses, physiotherapists and other members of the multidisciplinary team be familiar with this type of support because the clinical outcomes of patients on extracorporeal membrane oxygenation are directly related to the experience of the center in the management of these patients.
